# Feather Defects in a Juvenile Common Swift (*Apus apus*) Associated with a Circovirus Infection

**DOI:** 10.3390/vetsci12121117

**Published:** 2025-11-24

**Authors:** Marko Legler, Kristin Heenemann

**Affiliations:** 1 Department of Small Mammal, Reptile and Avian Medicine and Surgery, University of Veterinary Medicine Hannover, Foundation, Bünteweg 9, 30559 Hanover, Germany; 2 Institute of Virology, Centre for Infectious Diseases, Faculty of Veterinary Medicine, Leipzig University, An den Tierkliniken 29, 04103 Leipzig, Germany; kristin.heenemann@vetmed.uni-leipzig.de

**Keywords:** swift, virus infection, *Apodidae*

## Abstract

The common swift is the most observed swift species in Germany. During the summer months in urban areas, juvenile swifts in need of help are frequently cared for in wild bird rescue centers. Providing adequate housing and veterinary treatment for hundreds of juvenile swifts can be very challenging for non-specialized organizations and contact with other bird species is often unavoidable. The knowledge about the transmission of infectious diseases between individuals of this bird species specialized in an airborne life and of other bird species is limited. In this presented case a wild juvenile common swift was hand-reared for about 17 days and was noticed to have increasing feather abnormalities in a wildlife rescue center with close proximity to other wild birds. At the time of the investigation, the swift was of an age of about 35 days, and in good physical condition. The affected body, wing, and tail feathers showed retentions of feather sheaths, malformation of the feather vane, and circumferential constrictions with feather loss. The virological examination of the dystrophic feathers revealed a positive result for the circovirus and a negative result for the polyoma-consensus-nested PCR. The detected sequence of the circovirus showed the highest identity in 300 base pairs of 83.11% to a Canary circovirus isolate in NCBI Blast analysis. In addition to a transmission between common swifts during the nestling phase, other wild bird species can be discussed as a possible source of the infection.

## Simple Summary

The common swift (*Apus apus*), a widespread wild bird in Germany, can reach high population densities in urban areas. For example, the Hanover region hosts approximately 800 to 1200 breeding pairs. The common swift is known for its unique lifestyle, spending most of the year in continuous flight, interrupted only during the breeding season. During extreme weather conditions, such as heat waves, many juvenile swifts in need of help are brought to wildlife rescue centers and require veterinary care and rehabilitation. However, many aspects of their veterinary treatment remain insufficiently understood. Little is known about viral infections and their transmission in swifts. This case report describes a feather disease of a hand-reared juvenile common swift (*Apus apus*) housed in a wildlife rescue center in the Hanover region. The swift was referred to the clinic due to progressive feather abnormalities. A circovirus was identified from the abnormally growing feathers using circovirus-consensus nested PCR and was considered as the probable cause for the swift’s condition. To our knowledge, this is the first report of a circovirus infection in a swift species.

## 1. Introduction

The common swift (*Apus apus*) represents the most frequently observed species of the order Apodiformes in Germany. As a long-distance migratory bird, it undertakes annual migrations across Europe to spend the non-breeding season in sub-Saharan Africa, typically from September to May. During this period, common swifts remain airborne for over ten months per year, exhibiting highly specialized aerial behavior [1,2,3,4,5,6,7]. Urban environments can support substantial breeding populations, with national estimates ranging between 215,000 and 395,000 breeding pairs in Germany [8,9]. In the summer months, common swifts are regularly admitted to wildlife rescue centers, particularly juveniles. Admission rates tend to increase significantly during periods of extreme weather, such as heatwaves or extended cold periods, during which several hundred individuals may require care and rehabilitation [10,11]. The housing and appropriate care of large numbers of individuals of this avian species, which has specific biological and husbandry requirements, can pose considerable challenges. This is particularly the case in non-specialized wildlife rescue centers, where contact with other bird species is often unavoidable. In this context, current knowledge regarding infectious diseases in swifts and the potential transmission of pathogens from other avian species to common swifts remains limited [10,12,13]. Circovirids are small icosahedral virions of 20 to 25 nm with a small circular single-stranded DNA genome. The genome encodes for two proteins, the replication-associated protein and the capsid protein. Within the family *Circoviridae*, the Circovirids are classified into two genera, *Circovirus* and *Cyclovirus* [14,15,16,17,18]. Viruses of the genus *Circovirus* are common pathogens in birds worldwide [18]. Particularly, the beak and feather disease virus (BFDV) is well known and examined in parrots in captivity and the wild [14,19,20]. However, circoviruses have also been detected in other wild bird species, including, for example the BFDV in the rainbow bee-eater (*Merops ornatus*) [21], the pigeon circovirus (PiCV) in the Eurasian collared-dove (*Streptopelia decaocto*) and other wild pigeons [22], the goose circovirus (GoCV) in different species of wild geese [23], the duck circovirus (DuCV) in wild ducks [24,25,26], the gull circovirus (GuCV) in the herring gull (*Larus argentatus*) [27], the swan circovirus (SwCV) in the mute swan (*Cygnus olor*) or the starling circovirus (StCV) in European starlings (*Sturnus vulgaris*) and spotless starling (*Sturnus unicolor*) [28,29]. Circovirus species have recently been identified in Adélie penguin (*Pygoscelis adeliae*; PenCV) [30] American wigeons (*Mareca americana*; WigFec Circovirus 1 and WigFec Circovirus 2) [31], little bittern (*Ixobrychus minutus*; BitternCV), a European bee-eater (*Merops apiaster*; Bee-eaterCV) [32] and a tawny owl (*Strix aluco*; ToCV) [33]. Circoviruses have also been detected in other members of Passeriformes, particularly in different species of corvids and the blackbird (*Turdus merula*) [34]. In captivity, Circoviruses are also widespread among finches, e.g., the canary circovirus in Atlantic canary (*Serinus canaria*; CaCV), the finch circovirus in Gouldian finch (*Erythrura gouldiae*) and the zebra finch circovirus in *Taeniopygia guttata* [34,35,36,37]. However, there are little information’s about the circulation of circovirus infections in the wild bird populations and the expected pathology associated with such a viral infection [14,18,34]. Especially for swifts worldwide, there is no information available in the literature. Among avian hosts, circoviruses are characterized by a tropism for epithelial cells and lymphatic tissues [38]. Therefore, avian diseases caused by circovirus are mainly characterized by feather abnormalities and causally associated with immunosuppression. Immunosuppression can lead to growth retardation and wasting and increase susceptibility to secondary infections [38]. Morbidity and mortality rates can vary significantly from case to case depending on the bird species affected and the virus involved [38]. Vertical transmission of circoviruses via the egg has also been described [34].

The aim of this case presentation is to describe feather defects associated with a circovirus in the common swift (*Apus apus*).

## 2. Case Report

### 2.1. Clinical Case

A juvenile common swift with a history of progressively developing feather defects was presented for clinical examination by a wildlife rehabilitator. At the time of presentation, the bird had been kept in a wildlife rescue center for 17 days. During a period of warm weather, the bird had fallen out of the breeding site, but showed no changes in its feather growth at this time and was classified as healthy. The animal was kept in its own box in a room with other juvenile swifts and other different wild bird species with a frequently changing stock. The bird was on a diet with steppe crickets supplemented two times a week with Korvimin ZVT (WDT, Garbsen, Germany). Vitamin B was supplemented every 10 days sub cutaneous by the attending veterinarian. The loss of feathers has already been observed for a week at the time of the investigation.

**Figure 1:**
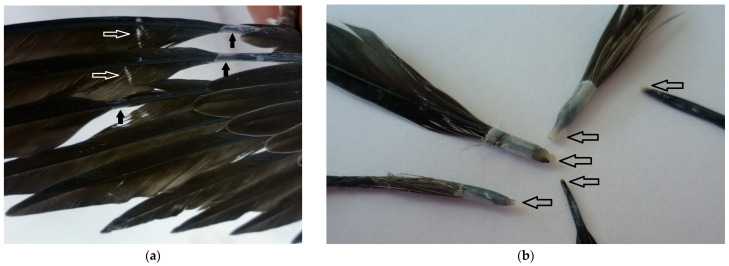

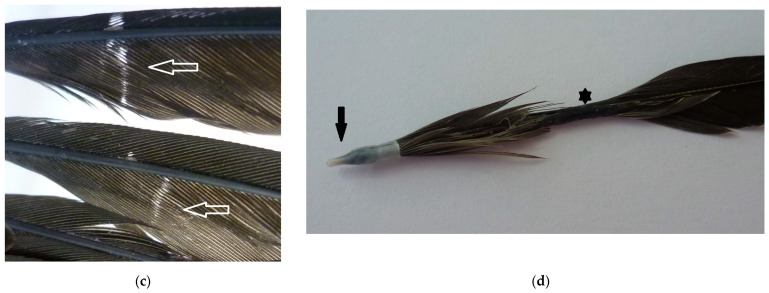
Feather defects of a juvenile common swift associated with a circovirus infection: (**a**): destruction of the closed wing surface due to disturbed feather growth of the primaries with persistent feather sheaths (black arrow) and fault bars (white arrow); (**b**): broken feathers of different body regions with typical circumferential constrictions (black arrow); (**c**): fault bars as a sign of growing disorders of the affected feathers (arrow); (**d**): fallen feather with a broken feather shaft due to a circumferential constriction (black arrow) and a visible malformation of the feather vane in the areas of the persistent feather sheaths (black star).

At the time of presentation, the juvenile swift was about 35 days old and in good physical condition with 36 g body weight. The development corresponded to the age of the common swift, and the bird showed normal begging behavior. The main findings were disturbances in feather development. Body feathers as well as wing, primaries and secondaries, and tail feathers were affected. The affected growing primaries and tail feathers showed retention of feather sheaths. A malformation of the feather vane was recognizable in the areas of the pathologic remaining feather sheaths, described as fault bars or “hunger streaks”. Feathers stopped the crowing, showed circumferential constrictions and fractures of the feather shafts and fell out of the feather follicles (Figure 1). Some feathers showed hemorrhage within the pulp cavity.

In order to rule out a viral infection, fresh growing feathers of the shoulder feathers and lost feathers were sampled and examined in a polyomavirus and a circovirus family-specific consensus-nested PCR [28,39].

The bird was euthanized due to the already heavily destroyed plumage at the time of presentation, and the poor prognosis for reintroduction into the wild, and the suspected viral infection with unfavorable prognosis for recovery. Unfortunately, we were not allowed to examine the animal further.

In addition, swifts without feather defect and with no known contact with other wild or diseased birds in the region of Hanover were screened for circo- and polyomaviruses. A total of 18 adult and 17 juvenile swifts, aged between 25 and 40 days, were euthanized due to severe injuries that precluded the possibility of successful rehabilitation. Feather samples collected postmortem were used for the virological examination

### 2.2. Virological Examination

The feathers were stored at 4 °C for further molecular diagnostic investigations. The DNA was extracted from a blood keel using the DNeasy Blood & Tissue Kit (Qiagen, Hilden, Germany). In order to investigate for viruses of the polyomavirus and circovirus families, family-specific consensus-nested PCRs were performed as described previously [28,39]. In the first-round of the Circovirus family-specific PCR, a product of approximately 600 bp was generated using the primers Cv-s (5′-AGAGGTGGGTCTTCACNHTBAAYAA) and Cv-as (5′-AAGGCAGCCAC-CCRTARAARTCRTC). The reaction mixture, with a total volume of 25.0 µL, consisted of the following components: 5.0 µL of 10× Pfx Amplification Buffer, 0.75 µL of dNTPs (10 mM each), 0.5 µL of MgSO_4_ (50 mM), 0.75 µL (10 µM) of each primer, 14.85 µL of DEPC-treated water, 0.4 µL of Platinum^®^ Pfx DNA Polymerase (Thermo Fisher Scientific, Waltham, MA, USA), and 2.0 µL of plasmid DNA or DNA (≤100 ng).

The thermal profile for the PCR product’s production was as follows: an initial denaturation at 94 °C for 5 min, followed by 35 cycles of 94 °C for 15 s, 60 °C for 30 s, and 68 °C for 40 s, and a final elongation at 68 °C for 2 min.

Subsequently, a second PCR was performed using the internal primers Cn-s (5′-AGCAAGGAACCCCTCAYYTBCARGG) and Cn-as (5′-ACGATGACTTCNGTCTT-SMARTCACG). The reaction mixture for this nested PCR, with a total volume of 25.0 µL, consisted of the following components: 5.0 µL of 10× Pfx Amplification Buffer, 0.75 µL of dNTPs (10 mM each), 0.5 µL of MgSO4 (50 mM), 0.75 µL (20 µM) of each primer, 14.85 µL of DEPC-treated water, 0.4 µL of Platinum^®^ Pfx DNA Polymerase, and 2.0 µL of a 1:10 dilution of the first PCR product.

The thermal profile used to generate the 350 bp PCR product was an initial denaturation at 94 °C for 5 min, followed by 35 cycles of 94 °C for 15 s, 60 °C for 30 s, and 68 °C for 20 s, with a final elongation at 68 °C for 2 min.

The resulting PCR products were analyzed on a 1.5% agarose gel alongside a 100 bp DNA ladder (Thermo Fisher Scientific, Waltham, MA, USA). The gel was stained with ethidium bromide and viewed under UV light. The polyomavirus family-specific PCR was performed using the same protocol as for circoviruses, with primer combination PVs (5′-CCAG-ACCCAACTARRAATGARAA) and Pvas (5′-AACAAGAGACACA-AATNTTTCCNCC) used in the first round and primer combination Pns (5′-ATGAAAATGGGGTTGGCCCNCTNTGYAARG) and Pnas (5′-CCCTCATAAACCCGAACYTCYTCHACYTG) used in the second round. After purification with the GenJet PCR Purification Kit (Thermo Fisher Scientific, Waltham, MA, USA), the positive PCR products were sent to Microsynth Seqlab GmbH (Göttingen, Germany) for sequencing. The Sanger method was applied for sequencing using the internal primers of the circovirus consensus nested PCR. To obtain the full genome, we also employed inverse PCR and rolling circle amplification (RCA) with both degenerate and specific primers [29,40]. The specific primers used for this process were designed based on the initial partial sequence obtained from the consensus PCR. Sequence quality control and editing were performed using the GENtle program (Version 1.9.0; Magnus Manske, University of Cologne, Germany). The identity of the processed sequences was confirmed by using the Basic Local Alignment Search Tool of the National Center for Biotechnology Information (NCBI; https://blast.ncbi.nlm.nih.gov/Blast.cgi, accessed on 7 April 2025). Phylogenetic analysis and the construction of phylogenetic trees were conducted using the software MEGA 12 [41,42]. The sequences were aligned within MEGA using the ClustalW algorithm. Phylogenetic relationships were subsequently determined using the Maximum Likelihood method, based on Kimura 2-parameter model [43]. The statistical support for the tree topology was evaluated through a bootstrap analysis with 1000 replicates.

#### 2.2.1. Results of the Virological Examination

The virological examination of the dystrophic feathers revealed a positive result for the circovirus-consensus-nested PCR and a negative result for the polyomavirus-consensus-nested PCR. Molecular characterization of the isolate yielded a partial sequence (349 bp) that was deposited in the NCBI-GenBank database under accession no.: PV416848. The NCBI Blast analysis showed the highest identity in 300 base pairs (bp) of 83.11% to a Canary circovirus isolate (NCBI accession no.: NC 003410.1).

No circo- or polyomaviruses were detected in feather samples from adult and juvenile common swifts without feather abnormalities and no history of staying at a wildlife rescue center.

#### 2.2.2. Phylogenetic Analysis

**Figure 2:**
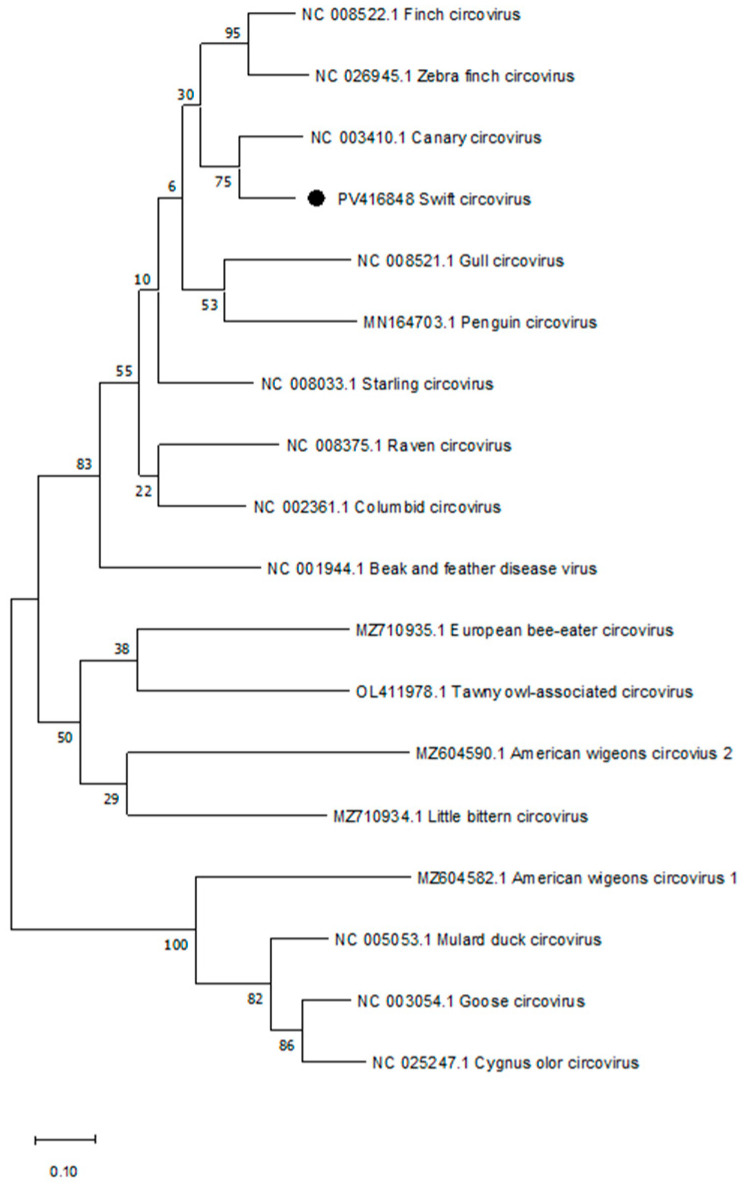
Phylogenetic analysis of partial swift circovirus and their relationship to 17 complete avian circovirus genomes. The sequence of the replicase gene (349 bp) amplified in this study from a common swift (NCBI Acc.-No.: PV416848, high-lighted with a black circle) was compared with 17 complete avian circovirus genome sequences from the GenBank. The evolutionary history was inferred by using the Maximum Likelihood method and Kimura 2-parameter model. The scale bar in the phylogenetic analysis shows how many substitutions per nucleotide position have taken place on average to explain the observed sequence differences.

For the phylogenetic analysis, we used a comprehensive set of complete avian circovirus genome reference sequences. This set included 17 available reference sequences of avian circoviruses from various hosts from the NCBI-GenBank database. The partial replicase gene sequences of the circovirus from this study (349 nucleotides) and all reference sequences were aligned, and a phylogenetic tree was constructed, representing the tree with the highest log likelihood (−4949.16). The percentage of trees in which the associated taxa clustered together is shown next to the branches. Phylogenetic analysis showed that the circovirus sequence from this study clustered with the Canary circovirus isolate (NCBI accession no.: NC 003410.1). The sequence showed a considerable genetic distance from all other avian circovirus references used in the analysis (Figure 2).

**Figure 3:**
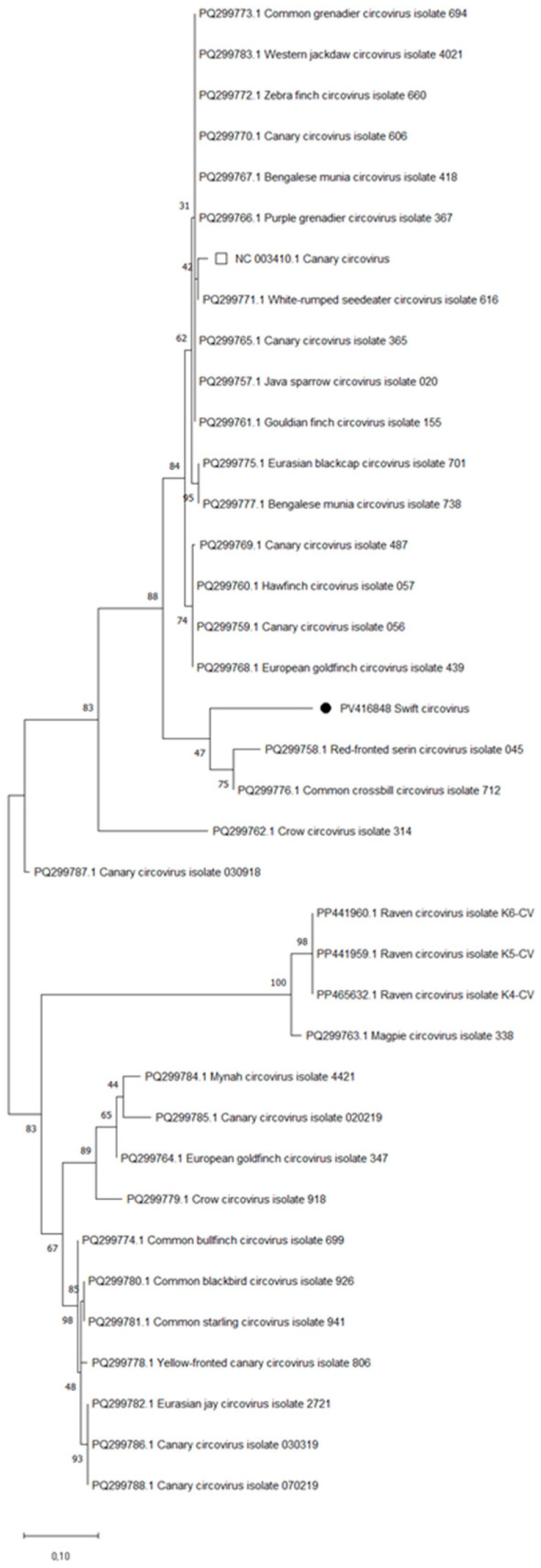
Phylogenetic tree showing the relationship between the swift circovirus swift (NCBI Acc.-No.: PV416848, high-lighted with a black circle) and various circovirus isolates from GenBank. Phylogenetic analysis was conducted using the Maximum Likelihood method with the Kimura 2-parameter model. The final dataset of this analysis comprised 37 nucleotide sequences with a total of 220 positions. To ensure high data quality, all positions with less than 95% site coverage were excluded. This means that alignment gaps, missing data, and ambiguous bases were allowed at any position only if they constituted less than 5% of the total data. The tree with the highest log likelihood (−1524.31) is displayed. The common swift’s sequence is marked with a black circle. The canary circovirus reference genome sequence is indicated with an empty square. All other sequences are from the study by Ledwoń et al. (2025) [34]. The scale bar in the phylogenetic analysis shows how many substitutions per nucleotide position have taken place on average to explain the observed sequence differences.

To provide a comprehensive analysis, we also included 35 recently published partial sequences [34] from various hosts with a focus on finches and other passerines in a separate phylogenetic reconstruction to ensure our analysis was as state-of-the-art as possible. This analysis, shown in Figure 3, demonstrates that the swift circovirus sequence (PV416848) groups with the sequences of a captive red-fronted serin (*Serinus pusillus*; PQ299758.1; Red-fronted serin circovirus isolate 045) and a common crossbill (*Loxia curvirostra*; PQ299776.1; Common crossbill circovirus isolate 712).

## 3. Discussion

The presented case of a juvenile common swift with feather loss associated with a circovirus is to our knowledge the first detection of a circovirus infection in a swift species [14,15,16,17,18]. Feather defects with circumferential constrictions were the main clinical indication of a possible circovirus infection and were the clinical focus in our case. The feather growing abnormalities that were observed in the juvenile common swift correspond to the disease symptoms in other bird species in connection with a circovirus, particularly in psittacines and especially budgerigars with the BFDV virus [19,20,21]. Similar to budgerigars with BFDV, it can be assumed that the age of a swift also has an influence on the severity of the viral disease [14,19]. The growth of the feathers in the juvenile swift probably also favored the typical feather loss. However, circovirus infections in finches, e.g., in canaries, cause mainly an increase in morbidity and mortality, but not feather defects [34,37,44]. In the swift case, there were no indications of a disturbed general condition in our examinations. The swift was in good nutritional condition with normal begging behavior. Unfortunately, immunocompetence could not be investigated further in this case though immunosuppression by circoviruses is described for other species, such as canaries, pigeons, ducks and geese [19,25,28,37,44].

The analysis of the partial sequence of the detected circovirus in the common swift showed the highest identity to a CaCV isolate and other circoviruses isolated from finches. Viruses are classified into separate species if their genomes share less than 80% genome-wide pairwise sequence identity with the classified family members [43]. This requirement is not fulfilled in our case, but we were only able to examine a partial sequence. Further research on circoviruses in swifts is essential to clarify this matter and to illustrate the spread of circoviruses in the common swift population.

Our investigations do not clarify whether the detected virus is a species-specific pathogen of the common swift. If this is the case, natural transmission could potentially occur within the nest during the breeding season. The transmission of circovirus is referred to as oral, intranasal and intra-cloacal routes [19] and the direct contact between the juveniles and the adults in the rearing period allows an easy transmission of the virus in the swift. A vertical transmission of the circovirus via the eggs should also be considered in swifts as a possible transmission route to the juvenile birds [34]. Outside the breeding season, transmission between swifts should be very difficult due to their specific aerial lifestyle. However, the clinical picture with a dystrophic feather loss as described in the presented case is very rare in swifts. Furthermore, it can be assumed that a viral infection causing feather defects would be subject to strong selective pressure and, therefore, is unlikely to occur in swifts. It is therefore quite possible that the swift in our case may have been infected with a circovirus from another bird species and developed a species-specific disease. The analysis of the partial sequence and its close phylogenetic relationship to circoviruses from finches suggest a potential host-jump or a shared reservoir between different bird species. This would be supported by the fact that the bird was kept in the same room with other bird species during the hand rearing. Natural infections between different wild birds could result from the fight for nesting sites and the use of the same nesting site [7]. Direct contact of this kind has been documented between swifts and house sparrows or starlings [7]. In the literature, the incubation period for a circovirus infection is reported to range from a few weeks to several years, with feather defects becoming apparent as early as three weeks after infection [19]. For swifts, however, no data are available. However, according to information in the literature for other bird species [19], it appears that the feather defects can develop after infection in the wildlife rescue center during the period of hand-rearing. Whether natural infections occur in the wild should be investigated in future studies on the prevalence of circoviruses in swifts. The absence of circovirus infection in the other juvenile and adult swifts we examined does not necessarily reflect the true prevalence within the population of the common swift. However, it does indicate that circoviruses are not ubiquitous subclinical within this species. The Hanover area has a population of 800 to 1200 breeding pairs [45].

The potential link between a newly identified feather disorder, termed Paper-Shaft-Syndrome, and a viral etiology remains entirely unexplored [46]. Consequently, further research into the occurrence of circoviruses in common swifts and other swift species, in relation to various diseases, is essential to identify potential correlations.

## Data Availability

The original contributions presented in this study are included in the article. Further inquiries can be directed to the corresponding author.
